# Supplementary material for: Middle through late Holocene long-distance transport of exotic shell personal adornments in Central West Patagonia (southern South America). The archaeomalacological assemblage of Baño Nuevo 1
Source: PLoS One. 2024 May 24;19(5):e0304454. doi: 10.1371/journal.pone.0304454 (PMC11125563; doi:10.1371/journal.pone.0304454)
Supplement: Attachment — Submitted filename: renamed_9d076.docx [file pone.0304454.s001.docx]

Bibliografía recomendada

Aportes teóricos

Lazzari, M. 1999. Objetos viajeros e imágenes espaciales: las relaciones de intercambio y la producción del espacio social. Revista do Museu de Arqueología e etnología. Suplemento, 371-385.

Lazzari, M. 1999. Distancia, espacio y negociaciones tensas: el intercambio de objetos en arqueología. En Sed non satiata, A. Zarankin & F. A. Acuto, Eds., pp. 117-151. Buenos Aires: Tributo.

Lazzari, M. 2005.Traveling objects and spatial images: exchange relationships and the production of social space. En Global archaeological theory: contextual voices and contemporary thoughts, P. Funari, A. Zarankin & E. Stovel, Eds., pp. 191-210. Nueva York: Kluwer Academic-Plenum Publisher.

Soto, C., Power, X., & Ballester, B. (2018). Circulación de objetos perforados de concha: Aportes para la interpretación de su rol en las relaciones sociales del desierto de Atacama entre los 6000-3500 AP. *Boletín del Museo Chileno de Arte Precolombino*, *23*(1), 51-69.

Complemento bibliografía regional

Gordillo, S., & Hajduk, A. (2022). BIENES SUNTUARIOS TRANSCORDILLERANOS EN EL SUR DE SUDAMÉRICA: EL CASO DEL CARACOL MARINOHOMALOPOMA. *Comechingonia*, *26*(3), 51-60.

Zubimendi, M., & Gordillo, S. 2022. Relaciones extrarregionales en Patagonia. Discusión a partir del análisis de artefactos foráneos elaborados sobre el caracol terrestre Mtegalobulimus. *Comechingonia. Revista de Arqueología*, *26*(1), 69-88.

Sobre tipos y manufactura

Soto Rodríguez, C. 2019. “Objetos perforados”, asociaciones simbólicas y redes de circulación: reflexiones sobre las formas de intercambio en el periodo Formativo (1500 ac-500 dc) del desierto de Atacama, norte de Chile. *Chungará (Arica)*, *51*(4), 573-593. (objetos perforados con huellas de uso y fissurellas, ver Figura 4 c y e)

Soto, C. (2009). Desde el mar y la selva: usos simbólicos de los restos malacológicos en la fase Tilocalar, quebrada Tulan (3500-2500 ap). *Memoria de pregrado. Facultad de Ciencias Sociales, Departamento de Antropología, Universidad de Chile*. https://repositorio.uchile.cl/tesis/uchile/2009/cs-soto_c/html/index.html (ref. páginas 63 y 64, ver Figura 7)
